# MOF negatively regulates estrogen receptor α signaling *via* CUL4B-mediated protein degradation in breast cancer

**DOI:** 10.3389/fonc.2022.868866

**Published:** 2022-09-23

**Authors:** Xu Zhang, Yang Yang, Danyang Li, Zhen Wu, Haoyu Liu, Ziyan Zhao, Hongying Zhu, Fei Xie, Xiangzhi Li

**Affiliations:** ^1^Shandong Provincial Key Laboratory of Animal Cells and Developmental Biology, School of Life Sciences, Shandong University, Qingdao, China; ^2^School of Pharmacy, Binzhou Medical University, Yantai, China; ^3^Rehabilitation Center, Qilu Hospital, Cheelo College of Medicine, Shandong University, Jinan, China

**Keywords:** MOF, ERα, breast cancer, protein degradation, CUL4B, tumor suppression

## Abstract

Estrogen receptor α (ERα) is the dominant tumorigenesis driver in breast cancer (BC), and ERα-positive BC (ERα+ BC) accounts for more than two-thirds of BC cases. MOF (males absent on the first) is a highly conserved histone acetyltransferase that acetylates lysine 16 of histone H4 (H4K16) and several non-histone proteins. Unbalanced expression of MOF has been identified, and high MOF expression predicted a favorable prognosis in BC. However, the association of MOF with ERα and the regulatory mechanisms of MOF in ERα signaling remain elusive. Our study revealed that the expression of MOF is negatively correlated with that of ERα in BC. In ERα+ BC cells, MOF overexpression downregulated the protein abundance of ERα in both cytoplasm and nucleus, thus attenuating ERα-mediated transactivation as well as cellular proliferation and *in vivo* tumorigenicity of BC cells. MOF promoted ERα protein degradation through CUL4B-mediated ubiquitin–proteasome pathway and induced HSP90 hyperacetylation that led to the loss of chaperone protection of HSP90 to ERα. We also revealed that suppression of MOF restored ERα expression and increased the sensitivity of ERα-negative BC cells to tamoxifen treatment. These results provide a new insight into the tumor-suppressive role of MOF in BC *via* negatively regulating ERα action, suggesting that MOF might be a potential therapeutic target for BC.

## Introduction

As the most common malignancy for women, breast cancer (BC) represents around 30% of female cancers and becomes the second leading cause of cancer-related mortality in women worldwide (1, 2). BC is a highly heterogeneous cancer with differential expression of tumorigenic marker genes like estrogen receptor α (ERα, or simply ER) or human epidermal growth factor receptor 2 (HER2) (3). Among them, ERα-expressing tumors, namely, ERα-positive BC (ERα+ BC), arise in 60%–80% of BC cases (4). As a steroid hormone nuclear receptor, ERα can be bound and activated by estrogen 17β-estradiol (E2) and serves as a transcription factor for the transactivation of oncogenes, like c-Myc and cyclin D1, thereby promoting cell proliferation and tumor progression of BC (5–7). In the absence of E2 stimulation, inactive ERα interacts with molecule chaperone heat shock protein 90 (HSP90) and can be maintained in a stable conformation for ligand binding (8). After binding with E2, ERα undergoes dissociation from HSP90 and translocates into the nucleus for the transcriptional activation/repression of target genes that encourage BC cell survival and growth (8, 9).

Therefore, as the major tumorigenesis driver in BC, modulation of ERα expression and function plays indispensable roles in the progression and treatment of BC (10). For instance, hypermethylation of ERα promoter leads to ERα deficiency, whereas treatment with DNA demethylating reagents plus inhibitors for histone deacetylases (HDACs) would restore ERα expression and tamoxifen (TAM) sensitivity in ERα-negative BC (ERα− BC) cells (11–13). ERα co-activators CBP/p300, functioning as histone acetyltransferases (HATs), enhance H3K27ac for facilitating ERα-mediated transcriptional activity, whereas pharmacological inhibition of CBP/p300 by A-485 and GNE-049 could downregulate ERα to suppress oncogenic c-Myc and cyclin D1 expression and the proliferation of ERα+ BC cells (4, 14, 15).

MOF (males absent on the first), also known as lysine acetyltransferase (KAT) 8 or histone acetyltransferase 1 (MYST1), is a highly conserved histone acetyltransferase (HAT) that specifically acetylates lysine 16 of histone H4 (H4K16) as well as non-histone proteins such as protein 53 kDa (P53),interferon regulatory factor 3 (IRF3), and lysine-specific demethylase 1 (LSD1) (16–19). MOF vigorously involves in diverse biological processes, such as transcriptional regulation, DNA damage repair, cell growth and differentiation, stem cell development, and tumorigenesis (20–22). Unbalanced expression of MOF is frequently observed in various tumors, such as colorectal carcinoma, gastric cancer, renal cell carcinoma, ovarian cancer, hepatocellular carcinoma (HCC), medulloblastoma, and primary breast carcinoma (20, 21). In particular, MOF was identified to suppress epithelial-to-mesenchymal transition (EMT) *via* the acetylation of histone demethylase LSD1 in lung cancer and BC, and higher expression of MOF is correlated with favorable prognosis in these two cancers (19, 23, 24).

Because of the inhibitory effect of MOF in BC tumor invasion and the essential role of ERα in tumor promotion, we are interested in the association of MOF with ERα as well as the modulatory effects of MOF on ERα expression and function to exert its carcinostasis potential in BC. We herein reported that MOF is negatively correlated with ERα expression in BC. In ERα+ BC, MOF negatively regulated the expression and nuclear localization of ERα to inhibit ER-mediated transactivation as well as the growth and tumorigenicity of ERα+ BC cells. MOF overexpression promotes ERα protein degradation *via* Cullin 4b (CUL4B)–mediated ubiquitin–proteasome pathway and HSP90 hyperacetylation that disrupts the chaperone binding of HSP90 with ERα. On the other hand, inhibited MOF by knockdown or inhibitor MG149 restored ERα expression and enhanced TAM sensitivity in ERα− BC cells. Our study provide new insights into the prohibitory function of MOF on ERα action in BC, suggesting that MOF might be a potential therapeutic target for BC.

## Material and methods

### Cell culture and cell transfection

MCF7, T47D, MDA-MB-231, and HCC1937 cells were obtained from the American Type Culture Collection. Cells were cultured in Dulbecco's modified Eagle's medium (DMEM) or Roswell Park Memorial Institute-1640 (RPMI-1640) (Macgene, Beijing, China) with 10% fetal bovine serum (FBS) (LONSA SCIENCE, Shanghai, China) and maintained in a humidified atmosphere containing 5% CO_2_ at 37°C. Cells were transfected with specific plasmid by JetPRIME (Polyplus, Strasbourg, France) according to the manufacturer’s protocol. The BC tissue chip was purchased from Guge Biotechnology Company (Wuhan, China).

### Antibodies and reagents

Anti-MOF (sc-81765) and breast-cancer susceptibility gene 1 (BRCA1) (Santa Cruz, sc-6954) were obtained from Santa Cruz Biotechnology. Antibodies including CUL4A (14851-1), CUL4B (12916-1), glyceraldehyde-3-phosphate dehydrogenase(GAPDH) (60004-1-Ig), and Flag (66008-2) were purchased from Proteintech (Wuhan, China). Other antibodies were listed as follows: H4K16ac (Epitomics, EPR1004), ERα (Cell Signaling Technology, Inc (CST), #8644), Ki67 (Abcam, ab16667), murine double minute 2 (MDM2) (Wanleibio, WL01906), HSP90 (Sangon Biotech, D120009), HSP90 K294ac (Rockland, 600-401-981), and acetylated lysine (CST, #9441). Inhibitors including MG149, cycloheximide (CHX), and MG132 were purchased from MedChemExpress (MCE, Princeton, NJ, USA). TAM was purchased from Sigma. CHX, MG149, MG132, and TAM were dissolved in dimethyl sulfoxide (DMSO).

### Immunohistochemistry staining

Immunohistochemistry (IHC) staining was performed to detect the expression of MOF and ERα in BC tissue chips. Following deparaffinization and quenching of endogenous peroxidase, the tissue section was treated by deparaffinization and quenching of endogenous peroxidase and then subjected to antigen retrieval with sodium citrate buffer. Then, the section was incubated with 5% FBS and then incubated with ERα (1:100) and MOF (1:100) antibodies overnight at 4°C. After incubation with secondary antibody at 37°C, the section was subjected to staining by the DAB Detection Kit (Polymer) (GeneTech, Shanghai, China) and counterstaining with hematoxylin (Solarbio, Beijing, China) for the observation with a light microscope (Nikon, Tokyo, Japan). All slides were scored in an open discussion by two experienced pathologists, who were blinded to the outcome. Immunostaining was scored on the basis of the intensity score and quantity of positive cell score. Intensity score: negative, 0; weak, 1; moderate, 2; and intense, 3. Quantity of positive cell score: <5%, 0; 5%–25%, 1; 26%–50%, 2; 51%–75%, 3; and >75%, 4. The product of intensity score and quantity of positive cell score was used as the total score.

### Immunofluorescence staining

Cells were seeded onto coverslips in 24-well plate for growth to 70% cell confluence. Cells were fixed using 4% paraformaldehyde and blocked by 5% FBS and then subjected to incubation with the primary antibody for MOF or ERα (1:100) overnight at 4°C. After incubation with corresponding secondary antibody, cells were mounted with DAPI (4',6-diamidino-2-phenylindole) (C0060, Solarbio), and images were taken from a DP74 color fluorescence camera (Olympus, Tokyo, Japan)

### RNA extraction and qRT‐PCR

Total RNA was isolated using RNAiso Plus (TaKaRa, Kyoto, Japan). RNA was reverse-transcribed by the RevertAid First Strand cDNA Synthesis Kit (Thermo Fisher Scientific, Waltham, MA, USA). Quantitative reverse transcriptase PCR (qRT-PCR) was performed using the SYBR qPCR Mix (TOYOBO). GAPDH was used as an internal control. Primers for qRT-PCR were listed in **Supplementary Table 1**. Then, relative quantitation of gene expression was calculated using the 2^−ΔΔCT^ method.

### Western blotting

Total protein was extracted using the sodium dodecyl sulfate (SDS) lysis buffer (1% sodium dodecyl sulfate, 5% glycerol, 1 mM ethylenediamine tetraacetic acid (EDTA), 25 mM Tris, 150 mM NaCl, and 1 mM phenylmethylsulfonyl fluoride (PMSF)). Protein samples were separated by SDS-polyacrylamide gel electrophoresis (PAGE) and transferred to polyvinylidene fluoride (PVDF) membranes (Millipore, Bedford, MA, USA). After blocking with 5% non-fat milk powder and incubation with specific antibody at 4°C overnight, membranes were subjected to corresponding secondary antibody and then visualized by an ECL detection kit (Wanleibio, Dalian, China).

### Immunoprecipitation

Proteins were extracted from cells using BC-200 lysis buffer (20 mM 2-[4-(2-hydroxyethyl)piperazin-1-yl]ethanesulfonic acid (HEPES), 200 mM KCl, 10 mM β-mercaptoethanol, 1 mM EDTA, 10% glycerol, and 0.1% NP-40) containing protease inhibitor cocktail (APExBIO, Houston, TX, USA). Extracted proteins were immunoprecipitated by incubation with 1 μg of antibody and followed by binding with Protein A/G magnetic beads (Bimake, Shanghai, China). After washing with lysis buffer, proteins were extracted by SDS sample buffer and detected by Western blotting.

### Chromatin immunoprecipitation assay

Chromatin immunoprecipitation (ChIP) assay was conducted using the SimpleChIP Plus Sonication Chromatin IP Kit (Cell Signaling Technology). Cells were cross-linked by 1% formaldehyde followed by sonication. The immunoprecipitated DNA was analyzed by qPCR with specific primers listed in **Supplementary Table 2**.

### Cell proliferation and colony formation

For cell proliferation assay, cells were seeded into 96-well plate and then treated with 10 µl of CCK8 (Biosharp) per 100 µl of culture medium at specific time points. After incubation at 37°C for 4 h, the absorbance value was determined at 450 nm by a SPECTROstar Nano instrument for calculating cell proliferation curves. In the colony formation assay, cells were seeded in 6-cm culture dish (200 cells per dish) and culture in 37°C incubator for 10–14 days. After termination of culture, cells were fixed with methanol and stained by crystal violet. The colonies with more than 50 cells per colony were counted.

### Xenograft tumor growth

Female NSG mice aged 6–8 weeks were prepared. MCF7 cells (5 × 10^7^ cells) with stable MOF transfection or control vector were subcutaneously injected into one flank of each mouse, respectively. The tumor growth was observed every 2 days. After 3 weeks, mice were sacrificed, and tumors were taken out for size measurement. Tumors were fixed with 4% paraformaldehyde and followed by IHC staining. Animal experiments were performed with the approval from the Animal Research Ethical Inspection Form of Shandong University School of Life Sciences (SYDWLL‐2018‐19).

### Statistical analysis

Data were statistically analyzed using GraphPad Prism software (San Diego, CA, USA) and were shown as means ± S.D. in three independent experiments. A Chi-square test was applied for analyzing pathological data. One-way ANOVA analysis was performed for time-course studies, and Student’s *t*-test was applied for comparisons of two groups. P < 0.05 was considered to be statistically significant.

## Results

### The expression of MOF is negatively correlated with that of ERα in BC tissues and cells

**Figure 1:**
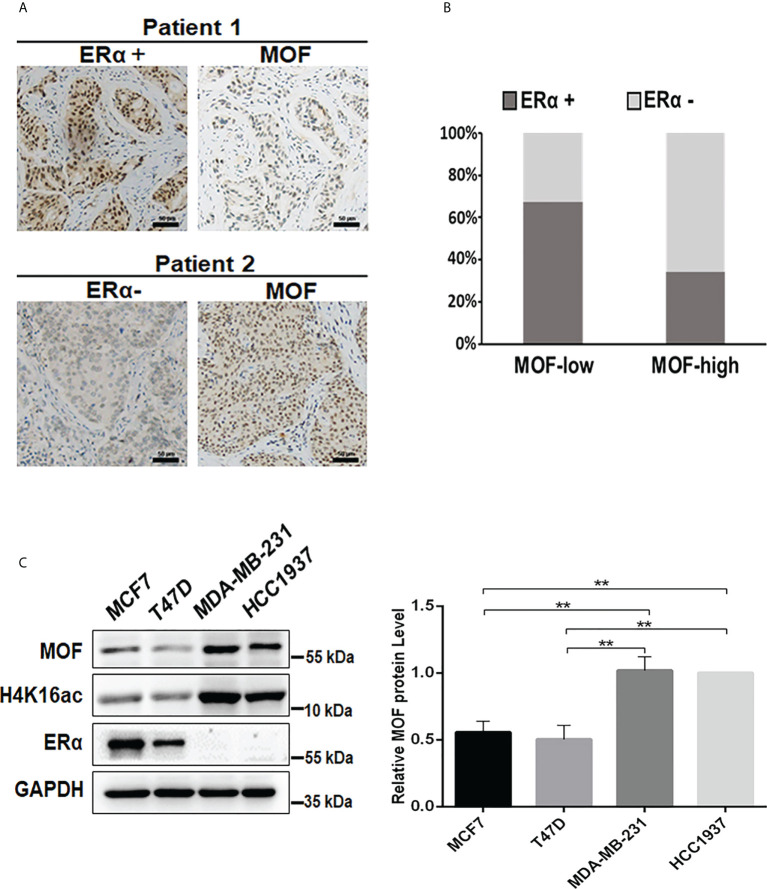
Analysis of the expression correlation of MOF and ERα in BC tissues and cells. **(A)** The protein abundance of MOF and ERα in BC tissue chip was determined by IHC staining, and the representative images were shown as high/positive and low/negative levels of MOF and ERα. Bar = 50 μm. **(B)** The staining results were quantified to demonstrate the correlation between MOF and ERα expression in BC tissues (n = 78). The staining was defined as high/positive (strong or moderate) and low/negative (weak or none) levels of expression. **(C)** The protein levels of MOF and ERα was analyzed by Western blot in BC cells, including ERα+ BC cells (MCF7 and T47D) and ERα− BC cells (MDA-MB-231). **P < 0.01 vs. control group.

MOF is reported as a critical suppressor in BC by inhibiting EMT and tumor invasion, suggesting a favorable prognosis (19); whereas ERα functions as the crucial oncogenic driver for the progression of BC (4). However, the relationship between the expression of MOF and ERα still remains elusive. To evaluate the correlation between MOF and ERα, we examined the protein expression level of MOF and ER in BC tissues from 78 patients. The immunohistochemical staining (IHC) results demonstrated the staining of MOF/ERα defined as either low/negative (weak or none) or high/positive (strong or moderate) based on the relative intensity of staining (**Figure 1A**). Statistical analysis of IHC results showed that around 64.5% of BC tumors with low MOF expression exhibited ERα-positive staining, whereas the majority (68.8%) of tumors with high MOF expression displayed ERα-negative staining, indicating that there is a negative correlation between MOF and ERα expression in BC tissues (**Table 1**, **Figure 1B**). Moreover, we examined the MOF and ERα expression in multiple BC cell lines. Western blot analyses revealed that the protein abundance of MOF with histone H4K16 acetylation exhibited a remarkably attenuated expression in ERα+ BC cells (MCF7 and T47D) compared with that in ERα− BC cells (MDA-MB-231 and HCC1937), and histone H4K16 acetylation also showed a similar pattern with MOF expression (**Figure 1C**). Taken together, these results suggested that MOF functions as a tumor suppressor in BC tumors and that the expression of MOF was negatively associated with that of ERα in BC tissues and cells.

**Table 1:** IHC analysis of MOF and ERα in BC tissues.

	ERα-positive	ERα-negative	Total
MOF-low	40 (64.5%)	22 (35.5%)	62
MOF-high	5 (31.2%)	11 (68.8%)	16
Total	45	33	78

Two-sided Pearson’s Chi-square test was conducted. P = 0.0163.

P < 0.05 was considered as statistical significance.

### MOF negatively regulates ERα protein level in ERα+ BC cells

**Figure 2:**
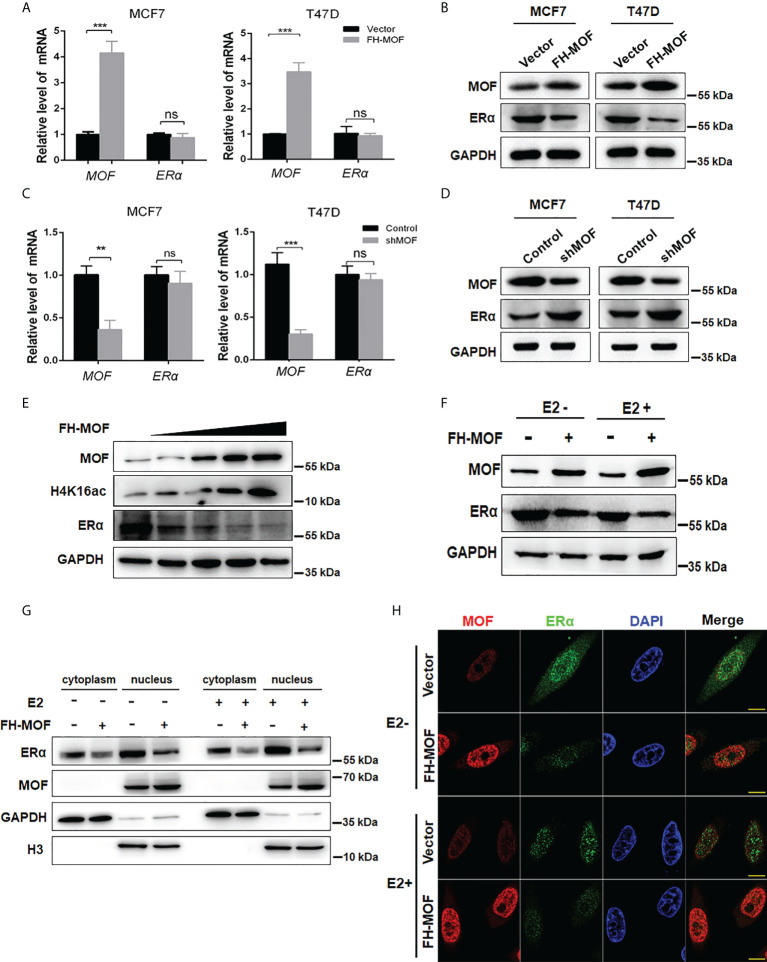
Effects of MOF overexpression on the expression of ERα in BC cells. **(A, B)** Effects of MOF overexpression on the steady-state mRNA levels and protein abundance of ERα in MCF7 and T47D cells were examined by qRT-PCR and Western blot assays. **(C, D)** MOF knockdown was performed by shMOF plasmid transfection to determine the expression of ERα at the mRNA and protein levels. **(E)** Increased doses of Flag-HA-MOF plasmid were transfected into MCF7 cells to examine the alteration trend of ERα protein levels. **(F)** Examination of the ERα protein level by MOF overexpression in the absence or presence of E2 treatment. After transfection with Flag-HA-MOF plasmid or empty vector as control for 48 h, MCF7 cells were treated with 10 nM E2 for 3h. **(G)** The protein expression of ERα by MOF in both cytoplasm and nucleus was determined by nuclear and cytoplasmic separation assay regardless of the presence of E2 in MCF7 cells. **(H)** Immunofluorescence staining assay was conducted to detect the expression of ERα in MOF-overexpressed MCF7 cells. Bar = 10 μm. ***P < 0.001 and **P < 0.01 vs. control group. ns, not significant vs. control.

To investigate whether and how MOF plays roles in the expression of ERα, plasmids of Flag-HA-MOF (for MOF overexpression) and pGPU6-shMOF (for MOF knockdown) were transfected into ERα+ BC cell lines (MCF7 and T47D), respectively. qRT-PCR analysis showed that the mRNA level of MOF was significantly upregulated or reduced in these cell lines, whereas the mRNA level of ERα had indistinguishable change (**Figures 2A, C**), suggesting that the expression of MOF did not regulate ERα expression at the transcriptional level. However, the protein abundance of ERα was obviously influenced by MOF overexpression or knockdown in a negatively regulatory manner (**Figures 2B, D**). Namely, both MCF7 and T47D cells transfected with Flag-HA-MOF plasmid (MOF overexpression) showed an increased amount of MOF and a decreased level of ERα protein (**Figure 2B**). Conversely, in the pGPU6-shMOF–transfected cells (MOF knockdown), the protein abundance of ERα was elevated (**Figure 2D**). In addition, when increased doses of Flag-HA-MOF plasmid were transfected into MCF7 cells, the ERα protein levels showed the corresponding downward trend with the gradually advanced expressions of MOF and H4K16ac (**Figure 2E**).

As a steroid hormone nuclear receptor, ER could be activated by estrogen 17β-estradiol (E2) (25). After binding with E2, homodimerized ER would translocate into the nucleus and functions as a transcription factor to regulate target gene transcription (25). Hence, we explored whether MOF-mediated regulation of ERα expression would be affected by E2 stimulation. After transfection with Flag-HA-MOF plasmid or empty vector as control for 48 h, cells were treated with E2 for 3 h. As depicted in **Figure 2F**, MOF overexpression could induce a significant reduction of ERα protein in the presence or absence of E2, suggesting that MOF downregulates the ERα protein level in an estrogen-independent manner. In addition, nuclear and cytoplasmic separation assay demonstrated that protein abundance of ERα in both cytoplasm and nucleus obviously decreased after MOF overexpression regardless of the presence of E2 (**Figure 2G**). Similar results were also observed by immunofluorescence staining that the distribution of ER in cytoplasm/nucleus was reduced in MOF-overexpressed MCF7 cells with or without E2 treatment (**Figure 2H**). These results suggest that MOF overexpression inhibited ERα protein levels in both cytoplasm and nucleus with or without E2 treatment.

### MOF prohibits the transactivation activity of ERα and cellular proliferation induced by estrogen and *in vivo* tumorigenicity

**Figure 3:**
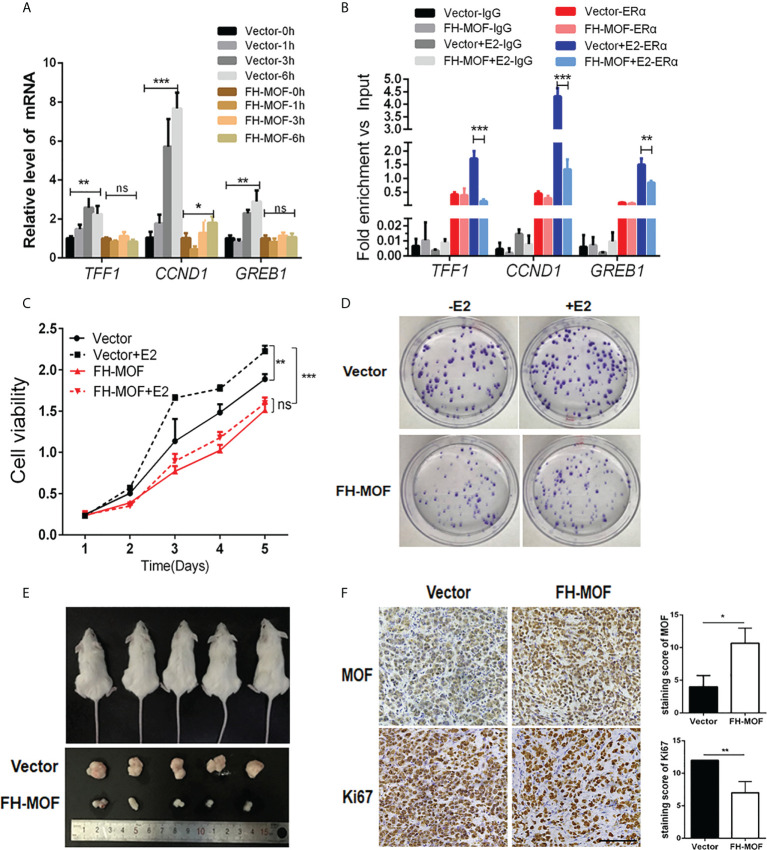
The inhibitory roles of MOF in ERα signaling, cellular proliferation, and tumorigenicity of BC cells. **(A)** The effects of MOF overexpression on ERα-mediated transcription activity of target genes (TFF1, CCND1, and GREB1) by qRT-PCR assay in E2-treated MCF7 cells. **(B)** The recruitment of ERα on the promoters of TFF1, CCND1, and GREB1 was analyzed by ChIP assay in MOF-overexpressed MCF7 cells under E2 stimulation. **(C)** Cell proliferation of MCF7 was prohibited by ectopic expression of MOF in CCK8 assay. **(D)** MOF restrained the colony formation ability of MCF7 cells whatever E2 is present. **(E)** Xenograft tumor-forming assay was conducted to determine the effect of MOF on in vivo tumorigenicity of MCF7 cells. MCF7 cells with stable MOF transfection or control were subcutaneously injected into one flank of each mice. Tumors were dissected from mice after 3 weeks of injection. **(F)** IHC staining (left) and staining score (right) showed the reduced expression of proliferation marker Ki67 in xenograft tumor tissue with MOF overexpression. Bar = 100 μm. ***P < 0.001, **P < 0.01, and *P < 0.05 vs. control group. ns, not significant vs. control. MOF restrained the colony formation ability of MCF7 cells, whatever E2 is present.

We next determined the effect of MOF on ERα-mediated transactivation upon E2 stimulation. MOF-overexpressed MCF7 cells were treated with E2 for specified incubation time, and the results showed that the mRNA expression of the three endogenous target genes (TFF1, CCND1, and GREB1) of ERα were significantly upregulated by E2 after 3 h of incubation in the control group cells. Whereas MOF overexpression abrogated this expression raise of ERα target genes by E2 (**Figure 3A**), suggesting that the transactivation abilities of ERα on target genes upon E2 treatment was prohibited by MOF overexpression. ChIP analysis further demonstrated that under the stimulation of E2, the recruitment of ERα at the promoters of TFF1, CCND1, and GREB1 was inhibited by ectopic expression of MOF (**Figure 3B**).

We further investigate the biological function of MOF in ERα+ BC cells. Moreover, stable cell lines with MOF overexpression were established by lentivirus infection of MCF7 cells, and CCK8 assay was performed to determine the functional role of MOF in BC cell proliferation. As shown **Figure 3C**, E2 stimulation significantly promoted cell proliferation of ERα+ BC MCF7 cells in the control group, whereas in MOF-overexpressed cells, this E2-stimulated raise was abolished, suggesting that MOF overexpression inhibited E2-induced proliferation of BC cells. In addition, the inhibitory effect of MOF on MCF7 cell prol9iferation occurred regardless of the presence or absence of E2 (**Figure 3C**), indicating that MOF prohibits cell proliferation of ERα+ BC cells in an E2-independent manner. In addition, MCF7 cells with shMOF transfection showed that MOF knockdown led to increased cell proliferation (**Figure S1A**). Colony formation assay further showed that MOF overexpression restrained the colony formation ability of MCF7 cells whenever E2 is present (**Figure 3D**). *In vivo* tumor formation experiments revealed that the size of tumor formed by MOF-overexpressed MCF7 cells was obviously smaller than that of control group cells (**Figure 3E**), indicating that MOF overexpression significantly impeded the growth of subcutaneous tumors formed by ERα+ BC cells in mice. In addition, IHC staining showed that, compared with the control tumor, reduced expression of proliferation marker Ki67 was observed in the tumor tissue with MOF overexpression (**Figure 3F**). Taken together, these results demonstrated that MOF overexpression prevented cell proliferation and tumorigenicity of ERα+ BC cells through the inhibition on ERα function.

### MOF promotes ERα protein turnover through ubiquitin–proteasome pathway

**Figure 4:**
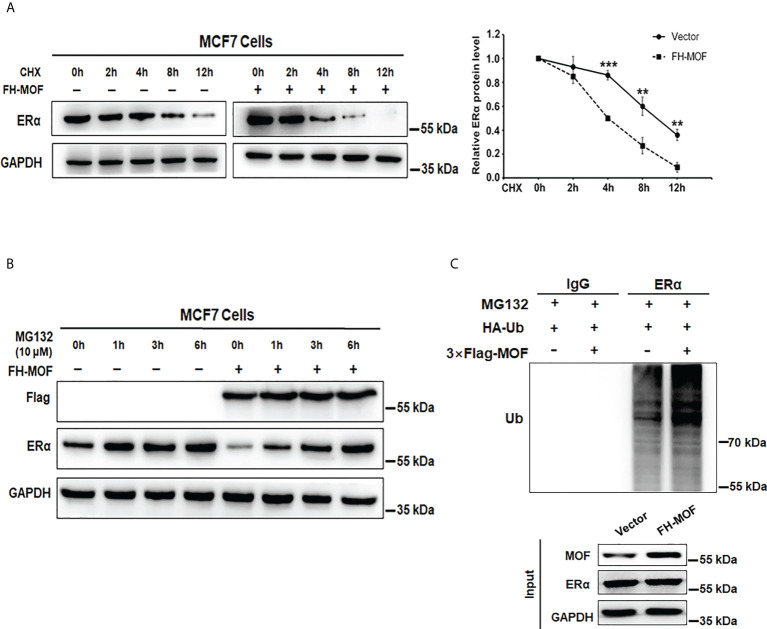
MOF promotes ERα protein degradation via ubiquitin–proteasome pathway. **(A)** CHX (10 μg/ml) assay was performed to examine the protein degradation of ERα under MOF overexpression. CHX (10 μg/ml) was applied for MCF7 cells with FH-MOF or control transfection, and cells were terminated at specified time points to calculate the half-life of ERα protein. **(B)** Proteasome inhibitor MG-132 could prevent ERα protein degradation induced by MOF overexpression. **(C)** Co-IP assay was performed in the presence of MG-132 to detect the polyubiquitin-conjugated ERα protein level in Flag-MOF–transfected cells. ***P < 0.001 and **P < 0.01 vs. control group.

MOF downregulates ERα protein abundance in MCF7 cells. We speculated that the protein stability of ERα might be affected by MOF for the negative effect on ERα expression. By using CHX, an inhibitor of protein translation, to block *de novo* protein synthesis, we found that ERα protein stability was attenuated by MOF overexpression (**Figure 4A**). Under CHX treatment, the degradation of ERα protein was overtly accelerated by MOF overexpression compared with the control group. The half-life of ERα was reduced down to around 4 h in the MOF-overexpressed MCF7 cells compared with that to around 9 h in the control group (**Figure 4A**). Conversely, we found that the application of MG132 (an inhibitor of proteasome function) could strikingly prevent ERα protein degradation induced by MOF. With the time extension of MG132 treatment, ERα protein expression increased gradually in MOF-overexpressed MCF7 cells and reached a similar level as the control group at 6 h (**Figure 4B**), suggesting that MOF-induced ERα protein degradation occurred by the proteasome pathway. Furthermore, polyubiquitination of ERα protein by MOF was observed in co-immunoprecipitation (Co-IP) assay. MOF overexpression strengthened the polyubiquitination of ERα, as shown by more intense ladder band of polyubiquitin-conjugated ERα protein in Flag-MOF–transfected cells (**Figure 4C**). In addition, MOF knockdown resulted in the abrogation of ERα polyubiquitination to promote ERα protein stability (**Figure S1C**). These results indicated that MOF promoted ERα protein degradation through ubiquitin–proteasome pathway.

### CUL4B is the functional E3 ligase involved in MOF-mediated ERα protein destabilization

**Figure 5:**
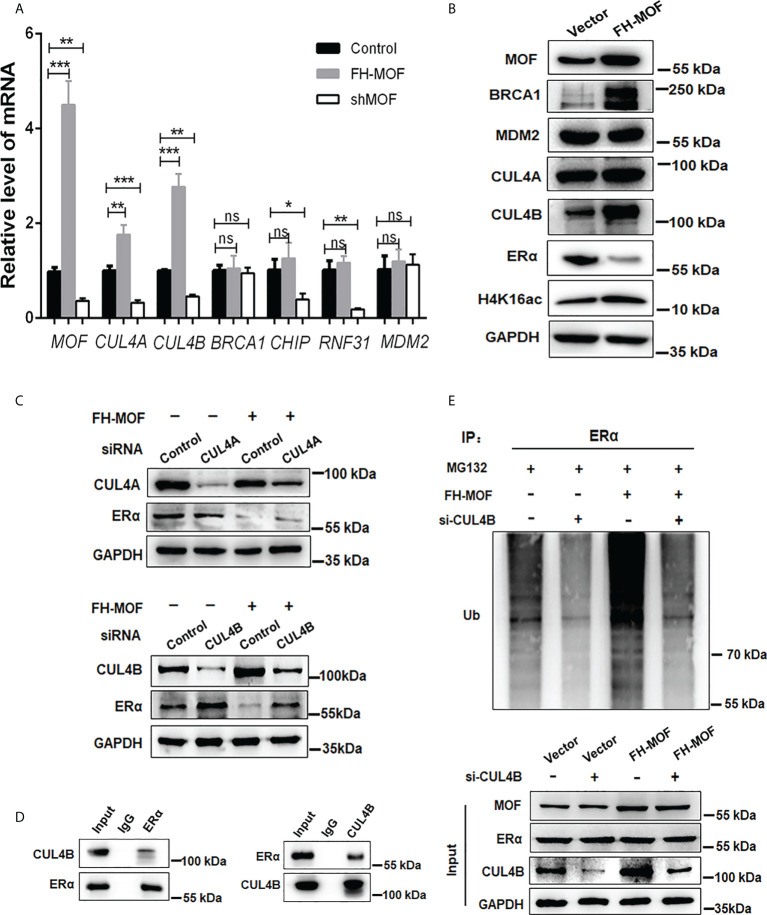
CUL4B is required for MOF-induced ERα protein degradation. **(A, B)** The mRNA and protein levels of candidate E3 ligases were determined by qRT-PCR and Western blotting in MCF7 cells with MOF overexpression. **(C)** Knockdown of CUL4B but not CUL4A could abrogate MOF-induced ERα protein degradation by WB assay. **(D)** Co-IP was performed to detect the physical interaction between MOF and ERα by using specific antibodies against the two proteins. **(E)** The levels of polyubiquitin-conjugated ERα was determined by Co-IP to examine the involvement of CUL4B in the ubiquitination and protein degradation of ERα. ***P < 0.001, **P < 0.01, and *P < 0.05 vs. control group. ns, not significant vs. control. .

We further explored the E3 ubiquitin ligase responsible for the MOF-induced ERα ubiquitination and degradation. Several E3 ligases like MDM2, CHIP, RNF31, and BRCA1 have been reported to trigger polyubiquitination of ERα for ubiquitin/proteasome-mediated proteolysis (26–29). Our RNA-seq raw data suggested a possible upregulation of CUL4A, which belongs to the Culling-Ring E3 ligase subfamily, in MOF-overexpressed MCF7 cells. After investigating the expression of several E3 ligase candidates in MCF7 cells harboring overexpression or knockdown of MOF, it was found that CUL4A and CUL4B can be positively modulated by MOF in a qRT-PCR assay (**Figure 5A**). In addition, the protein abundance of CUL4A and CUL4B could be upregulated by MOF overexpression (**Figure 5B**). To further confirm the involvement of CUL4A or CUL4B in MOF-mediated ERα degradation, cells were co-transfected with FH-MOF plasmids and CUL4A or CUL4B small interfering RNA (siRNA). It was demonstrated that blockage of CUL4B but not CUL4A could abrogate MOF-induced ERα protein degradation (**Figure 5C**). Moreover, Co-IP assay revealed that CUL4B and ERα proteins could physically interact with each other (**Figure 5D**). In addition, CUL4B knockdown abolished MOF-encouraged ubiquitination of ERα as revealed by the reduced amount of polyubiquitin-conjugated ER in CUL4B siRNA-transfected cells (**Figure 5E**). These results indicated that MOF promoted the ubiquitination and protein degradation of ERα *via* upregulated CUL4B functioning as an E3 ligase.

### MOF promotes HSP90 hyperacetylation to inhibit its chaperon association with ERα

**Figure 6:**
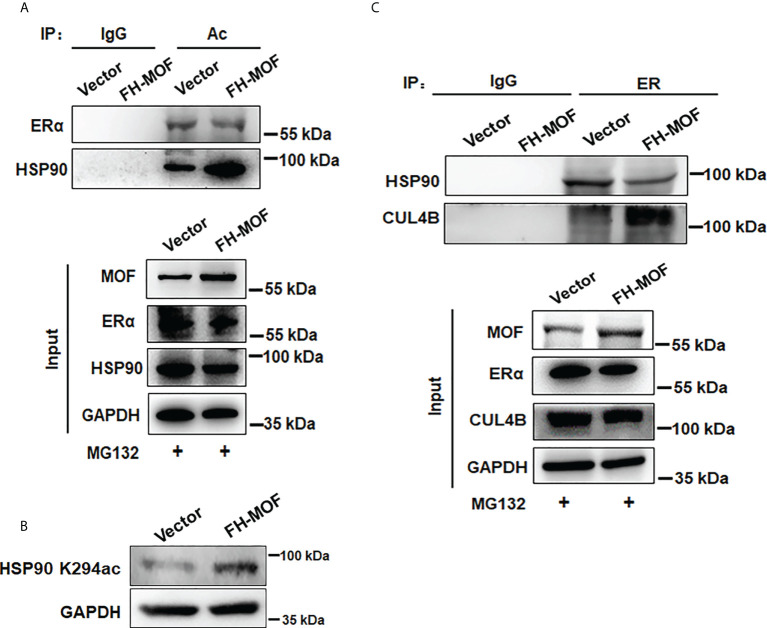
MOF promotes HSP90 hyperacetylation to inhibit its chaperon association with ERα. **(A)** The acetylation level of ERα and HSP90 was investigated by Co-IP using acetylated lysine antibody for IP and antibodies against ERα and HSP90 for Western blotting in FH-MOF–transfected MCF7 cells. **(B)** HSP90 K294 acetylation site was identified to be functioning in MOF-induced hyperacetylation of HSP90 by Western blotting assay using specific HSP90 K294ac antibody. **(C)** MOF overexpression enhanced the protein interaction between ERα and CUL4B but undermined the chaperon association of HSP90 with ERα in Co-IP assay.

Molecular chaperone HSP90 binds with ERα to maintain the conformational stability of ER for ligand binding and to protect ERα from protein degradation, whereas hyperacetylation of HSP90 inhibits its chaperone function for ERα (9). By Co-IP assay, it was shown that the acetylation level of HSP90 was overtly raised in MOF-overexpressed MCF7 cells, whereas the acetylation level of ERα was not obviously affected (**Figure 6A**). In addition, MOF knockdown also did not overtly affect the acetylation level of ERα but markedly decreased that of HSP90 (**Figure S1B**) It was further confirmed that MOF-induced hyperacetylation of HSP90 occurred through the K294 acetylation site (**Figure 6B**), which was reported to be determinant for the chaperone binding of HSP90 with its client proteins (30). Co-IP assay further revealed that when MOF was overexpressed in MCF7 cell, the interaction between HSP90 and ER was undermined, whereas more association of ER with CUL4B was observed instead (**Figure 6C**). Taken together, it is suggested that MOF enhanced the acetylation level of HSP90 at K294 site to attenuate the chaperone association of HSP90 with ERα, thereby liberating ERα to more interact with CUL4B for ubiquitin-mediated proteasomal degradation of ERα.

### Inhibition of MOF restores ERα protein abundance and increases TAM sensitivity in ERα− BC cells

**Figure 7:**
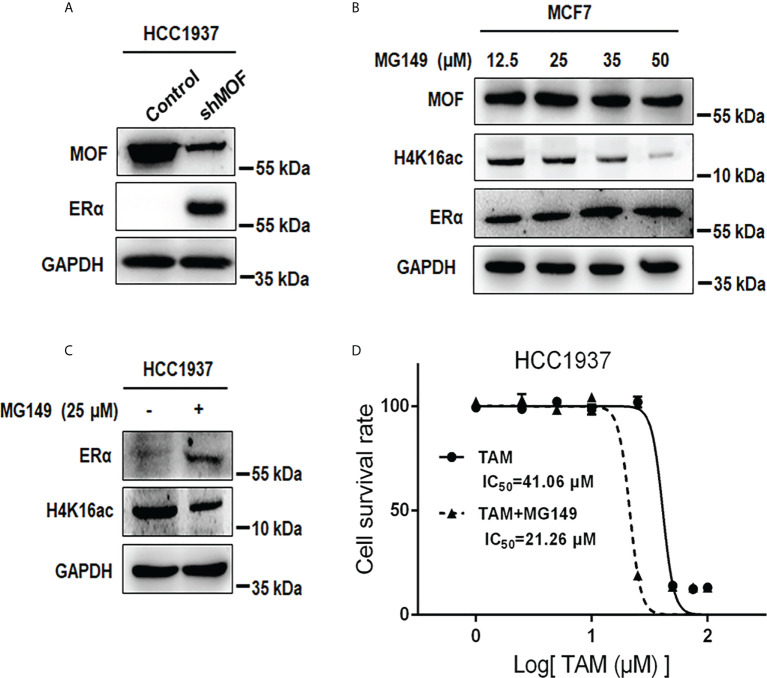
Effects of MOF inhibition on ERα protein expression and cell viability in ERα− HCC1937 cells. **(A)** MOF knockdown restored ERα protein abundance in ERα− HCC1937 cells transfected with pGPU6-shMOF plasmid as determined by Western blotting. **(B)** Different concentration of MOF inhibitor MG-149 was applied to examine its enhanced effect on ERα protein expression in MCF7 cells. **(C)** ERα protein expression was reactivated by 35 μM MG149 in ERα− HCC1937 cells. **(D)** Cell viability assay was performed to investigate the effect of MG149 on the sensitivity of ERα− HCC1937 cells to tamoxifen (TAM) treatment. IC_50_ was calculated to compare the combinatory effect of TAM and MG149 with TAM alone treatment for 24 h.

In addition to the negative regulation of MOF overexpression on ERα protein stability in ERα+ BC cells, we also examined the effect of MOF inhibition on ERα− BC cells. As shown in **Figure 7A**, knockdown of MOF in ERα-negative HCC1937 cells resulted in a recovery of ERα protein expression. As an HAT inhibitor, MG149 could inhibit MOF within a certain concentration range (47 ± 14 µM) because higher concentration would work on other histone acetylases (like 74 ± 20 µM for Tip60) (31, 32). First, the inhibitory effect of MG149 was verified in ERα+ MCF7 cells, where increased doses of MG149 could result in the raised abundance of ERα protein and reduced H4K16ac (**Figure 7B**). Then, treatment of 35 μM MG149 in ER− HCC1937 cells could obviously restore ERα protein expression similar as the effect of MOF knockdown (**Figure 7C**). Because reactivation of ERα expression could restore endocrine therapy sensitivity in patients with ERα− BC (12, 13), we further investigated the effect of MG149 on the sensitivity of ER− BC cells to TAM. Compared with the inhibition concentration IC50_50_ of TAM at 41.06 μM in HCC1937 treated with TAM alone for 24 h, IC_50_ was reduced to 21.26 μM with a combinatory treatment of TAM and MG149 for 24 h (**Figure 7D**), suggesting that MOF inhibitor MG149 could effectively improve the response of ERα− BC cells to TAM treatment by the restoration of ERα protein abundance.

## Discussion

ERα, encoded by the gene of estrogen receptor 1 (ESR1), is one of the major tumorigenic drivers in BC and uterine cancer (10, 33). ERα-expressing BC, also called luminal BC, accounts for more than two-thirds of patients with BC (10). Because of the full weight of ER functioning in fueling tumor behavior, post-translational modifications of ERα protein and/or epigenetic regulation of ESR1 gene have drawn much attention for their roles in the expression and activity of ERα for controlling the growth and tumorigenicity of cancer cells (34–36). MOF, functioning as a lysine acetyltransferase for the acetylation of H4K16ac as well as multiple non-histone proteins, is currently identified for its aberrant expression and playing regulatory roles in diverse cancers (37–39). For instance, MOF overexpression promoted the cell proliferation, migration, and drug resistance of lung non–small cell lung cancer cells (39), whereas the lack of MOF resulted in the hypoxia tolerance and multidrug resistance of HCC cells through upregulated hypoxia-inducible factor-1α (HIF-1α) (40). It was reported that in large cohort of patients with BC or lung cancer, high MOF expression showed a favorable prognosis (19, 23, 24), which is consistent with the demonstration in the present study that the expression of MOF is negatively correlated with that of ERα in BC tissues and cells. We unraveled that MOF overexpression downregulated ERα expression to inhibit the transactivation potential of ERα as well as the proliferation and tumorigenicity of ERα+ BC cells.

The reduced ERα expression by MOF overexpression occurred at the post-translational level *via* promoting ER protein degradation but not at the mRNA level. MOF-mediated ERα degradation requires the activation of CUL4B to speed up ERα protein turnover by the proteasome machinery. CUL4B belongs to the Cullin-Ring E3 ubiquitin ligase subfamily whose members were reported to participate in the proteolysis *via* catalyzing polyubiquitination of various substrates for proteasomal degradation and are implicated in the regulation of some pathological processes (41). For instance, CUL5 is responsible for IFN-gamma–induced proteasomal degradation of HER2 in BC, resulting in diminished cell growth and tumor senescence (42). In addition, CUL4B is responsible for long noncoding RNA Nron-mediated ERα protein stability in osteoporosis (43). Similar with these findings, our data showed that CUL4B destabilized ERα when MOF was overexpressed in BC cells because MOF promoted more expression and interaction of CUL4B with ERα for its polyubiquitination and degradation. As for the two Cullin 4 genes (CUL4A and CUL4B), they shared high identity of protein sequence (44) and possessed overlapping functions in certain scenario (45, 46), like in DNA damage response and polyubiquitination of p53 for degradation (47, 48). However, distinct roles for these two Cul4 proteins have also been revealed recently (49, 50). Accordingly, in our study, both CUL4A and CUL4B were positively regulated by MOF; nevertheless, only the knockdown of CUL4B could abrogate MOF-induced ERα protein degradation.

Apart from the role of CUL4B in MOF-induced ERα protein destabilization, our data also showed that the acetylation of HSP90 might be associated with the effect of MOF on ERα expression. As a molecular chaperone HSP90 interacts with ERα to maintain a stable conformation of ERα for ligand binding and being protected from degradation (8, 9). Previous studies have revealed that hyperacetylation of HSP90 induced by HDAC6 depletion or HDAC inhibitors would restrain the chaperone function of HSP90, thereby promoting the polyubiquitylation and proteasomal degradation of client proteins, like c-Raf, Akt, cyclin D1, and ERα, to evoke growth arrest and apoptosis of cancer cells (9, 51). Similarly, our data showed that MOF overexpression heightened the acetylation of HSP90 and thereby hampered the interaction between HSP90 and ERα, implying the contribution of MOF-induced hyperacetylation of HSP90 in the promotion of ERα degradation. Moreover, the acetylation of HSP90 K294 site was known to be essential for weakening the chaperone association of HSP90 with diverse client proteins such as ErbB2, mutant p53, HIF-1, and androgen receptor (30). In our study, HSP90 K294 site can be specifically acetylated by MOF overexpression, implicating that HSP90 K294ac might play a functional role in MOF-elicited dissociation of ER from HSP90 that results in ER protein instability. Taken together, MOF promoted the hyperacetylation of HSP90 to liberate ERα from the chaperone binding, and subsequently, more CUL4B was recruited to ERα for inducing ER polyubiquitination and proteasomal degradation. A similar scenario was demonstrated where inhibited HSP90 function would destroy the chaperone binding of HSP90 with mutant p53, thereby triggering the protein degradation of released mutant p53 *via* E3 ligases MDM2 and CHIP-mediated ubiquitin–proteasome pathway (52).

On the basis of its role in histone H4K16 acetylation, MOF serves as co-activator of nuclear factor–κB and androgen receptor for upregulating their transactivation capacity in prostate cancer (53, 54). Dimethylation of ERα by G9a could be recognized by MOF complex to induce transcriptional activation of ERα target genes (55). In addition, MOF-mediated acetylation of non-histone proteins plays essential roles in distinct cancer cells. For instance, MOF acetylates the histone demethylase LSD1 to impede its binding with epithelia genes for their transactivation, thus suppressing EMT and tumor progression in lung cancer and BC (19). MOF-mediated acetylation of HIF-1α causes the ubiquitination and degradation of HIF-1α to affect hypoxia susceptibility and drug resistance in HCC (40). On the contrary, MOF acetylates ERα to maintain ERα stability *via* reduced polyubiquitination, thus promoting ER signaling and inhibiting HCC progression (56). This discrepancy in ERα protein stability elicited by the same acetylase activity of MOF on non-histone proteins could be due to different functional characteristics of ERα caused by distinct cellular environment in diverse cancer types and various acetylation targets that MOF acts on.

At present, hormonal therapies have been extensively applied for the treatment of ERα+ BC, like aromatase inhibitors for suppressing estrogen synthesis and antiestrogens competing with estrogens for the interaction with ERα to hinder ERα signaling pathway, which are the most common cure strategies (57, 58). However, for congenital ERα-negative tumors and relapsed tumors losing ER expression after endocrine treatment, they would exhibited intrinsical or acquired resistance to hormonal therapies due to lack of ERα expression (3, 59). Hence, restored expression of lost ERα will be an effective strategy for the sensitivity recovery to endocrine treatment. On account of the negative correlation between MOF and ERα in BC, we further identified that inhibition of MOF by knockdown or inhibitor MG149 could enhance ER expression in BC cells. In particular, in ER-negative HCC1937 cells, recovered abundance of ER protein by MG149 would partially restore the sensitivity of BC cells to TAM treatment. Hypermethylation of ERα gene was reported to be an important cause of suppressed ERα expression (9, 11), and the combination of DNA demethylating agents with HDAC inhibitors would restore ERα expression and TAM sensitivity in ERα− BC cells (12, 13). However, on the other hand, other studies reported that pan-HDAC inhibitors induce HSP90 hyperacetylation to inhibit its binding to ERα and promote ERα degradation (9, 60, 61). Consequently, we provided that the functional role of MOF in ER expression is prone to be similar with the action of HDAC inhibitors through HSP90 acetylation. A high level of MOF in ERα− BC cells resulted in the instability of ER protein due to HSP90 hyperacetylation and loss of chaperone function, whereas MOF inhibition would abrogate the foregoing effects to restore ERα abundance and partial sensitivity to endocrine therapy.

In summary, we unraveled the inverse correlation between MOF and ERα in BC tissues and cells, and MOF overexpression promoted ERα protein degradation *via* CUL4B-mediated ubiquitin–proteasome pathway and HSP90 hyperacetylation that lead to the loss of chaperone binding of HSP90 with ERα, thus inhibiting transcriptional activation and cellular proliferation induced by estrogen and *in vivo* tumorigenicity of ERα+ BC cells. In addition, suppression of MOF restored ERα expression and increased the sensitivity of ERα− BC cells to TAM treatment. These findings highlight an essential role of MOF in modulating ER signaling in BC and rationalize MOF as a potential therapeutic target, like developing specific MOF activator for anti-ER treatment in ERα+ BC or combination therapy of MG149 with TAM for resistance amelioration in ERα− BC.

## Data availability statement

The original contributions presented in the study are included in the article/**Supplementary Material**. Further inquiries can be directed to the corresponding author.

## Ethics statement

The animal study was reviewed and approved by Animal Research Ethical Inspection Form of Shandong University School of Life Sciences.

## Author contributions

XL designed this work. XZ, YY, DL, ZW, HL, ZZ, HZ, and FX performed the experiments. XZ and YY analyzed the data. XZ, YY, and XL wrote this manuscript. All authors have reviewed and approved the manuscript.

## Funding

This work was supported by the National Key R&D Program of China (2016YFE0129200) and the National Natural Science Foundation of China (Nos. 31571321, 31171428, 71974113 and 81601337).

## Conflict of interest

The authors declare that the research was conducted in the absence of any commercial or financial relationships that could be construed as a potential conflict of interest.

## Publisher’s note

All claims expressed in this article are solely those of the authors and do not necessarily represent those of their affiliated organizations, or those of the publisher, the editors and the reviewers. Any product that may be evaluated in this article, or claim that may be made by its manufacturer, is not guaranteed or endorsed by the publisher.

## Supplementary material

Supplementary Figure 1 MOF knockdown increased cellular proliferation of MCF7 cells and abrogated polyubiqutination but not acetylation of ERα. (A) CCK8 assay showed that cell proliferation was increased in MOF knockdown MCF7 cells. (B) Co-IP assay was performed in the presence of MG-132 to detect polyubiquitin-conjugated ERα protein level in pGPU6-shMOF–transfected cells. (C) The acetylation level of ERα and HSP90 was investigated by Co-IP using acetylated lysine antibody in pGPU6-shMOF–transfected MCF7 cells. ** P < 0.01 vs. control group.
