# Supplementary figures and images for: MOF negatively regulates estrogen receptor α signaling via CUL4B-mediated protein degradation in breast cancer
Source: Front Oncol. 2022 Sep 23;12:868866. doi: 10.3389/fonc.2022.868866 (PMC9539768; doi:10.3389/fonc.2022.868866)

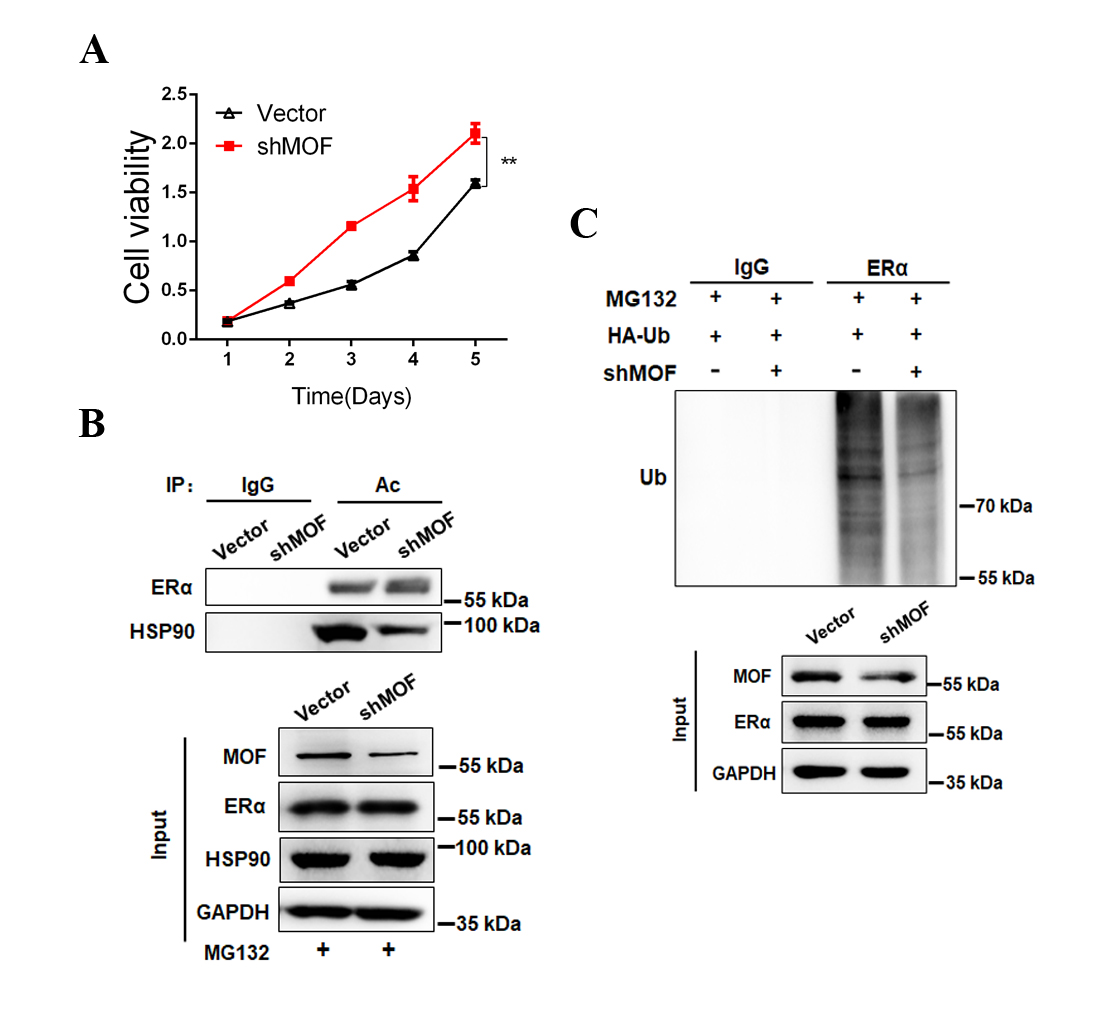

Supplement: Supplementary Figure 1 — MOF knockdown increased cellular proliferation of MCF7 cells and abrogated polyubiqutination but not acetylation of ERα. (A) CCK8 assay showed that cell proliferation was increased in MOF knockdown MCF7 cells. (B) Co-IP assay was performed in the presence of MG-132 to detect polyubiquitin-conjugated ERα protein level in pGPU6-shMOF–transfected cells. (C) The acetylation level of ERα and HSP90 was investigated by Co-IP using acetylated lysine antibody in pGPU6-shMOF–transfected MCF7 cells. ** P < 0.01 vs. control group. [file Image_1.jpeg]
